# Social Participation Patterns and Well-Being Among Older Adults: Findings From the United States, the UK, and China

**DOI:** 10.1093/geroni/igab046.1134

**Published:** 2021-12-17

**Authors:** Chenxin Tan, Yun Zhou, Bei Wu

**Affiliations:** 1 New York University, New York, New York, United States; 2 Department of Sociology, Peking University, Beijing, Beijing, China (People's Republic)

## Abstract

This study used Latent Class Analysis to examine patterns of social participation among older adults in the US, the UK, and China, from the three nationally representative surveys conducted in 2018-2019: The Health and Retirement Study, the English Longitudinal Study of Ageing, and the China Health and Retirement Longitudinal Study. Although the profiles of social participation were distinctively different, several common patterns were found: Comprehensive Participants, Occasional Participants, and Deficient Participants. It was estimated that less than 10% of older adults from these countries were extensively engaged in social participation. Seventy-seven percent of Chinese older adults were shown being “Deficient Participants”, and the percentages were 29% and 20% in the US and the UK, respectively. The findings showed positive associations of levels of participation with socioeconomic status and health. The magnitudes of these associations varied across the nations. Actions are needed to promote levels of participation for Chinese older adults.

